# Implementation and evaluation of a multisite drug usage evaluation program across Australian hospitals - a quality improvement initiative

**DOI:** 10.1186/1472-6963-11-206

**Published:** 2011-08-29

**Authors:** Lisa K Pulver, Angela Wai, David J Maxwell, Marion B Robertson, Steven Riddell

**Affiliations:** 1 School of Pharmacy, The University of Queensland, Brisbane, Queensland, Australia; 2 Australian Commission on Safety and Quality in Health Care, Sydney, New South Wales, Australia (formerly NPS - Better choices, Better health, Sydney, New South Wales, Australia; 3 Health Services Research and Effectiveness, NHS Quality Improvement, Edinburgh, Scotland (formerly New South Wales Therapeutic Advisory Group, Sydney, New South Wales, Australia; 4 Pharmacy Department, Royal Melbourne Hospital, Melbourne, Victoria, Australia and Victorian Drug Usage Evaluation Group, Melbourne, Victoria, Australia; 5 Ipsos Marketing - Health, Sydney, New South Wales, Australia (formerly NPS - Better choices, Better health, Sydney, New South Wales, Australia

## Abstract

**Background:**

With the use of medicines being a broad and extensive part of health management, mechanisms to ensure quality use of medicines are essential. Drug usage evaluation (DUE) is an evidence-based quality improvement methodology, designed to improve the quality, safety and cost-effectiveness of drug use. The purpose of this paper is to describe a national DUE methodology used to improve health care delivery across the continuum through multi-faceted intervention involving audit and feedback, academic detailing and system change, and a qualitative assessment of the methodology, as illustrated by the Acute Postoperative Pain Management (APOP) project.

**Methods:**

An established methodology, consisting of a baseline audit of inpatient medical records, structured patient interviews and general practitioner surveys, followed by an educational intervention and follow-up audit, is used. Australian hospitals, including private, public, metropolitan and regional, are invited to participate on a voluntary basis. De-identified data collected by hospitals are collated and evaluated nationally to provide descriptive comparative analyses. Hospitals benchmark their practices against state and national results to facilitate change. The educational intervention consists of academic detailing, group education, audit and feedback, point-of-prescribing prompts and system changes. A repeat data collection is undertaken to assess changes in practice.

An online qualitative survey was undertaken to evaluate the APOP program. Qualitative assessment of hospitals' perceptions of the effectiveness of the overall DUE methodology and changes in procedure/prescribing/policy/clinical practice which resulted from participation were elicited.

**Results:**

62 hospitals participated in the APOP project. Among 23 respondents to the evaluation survey, 18 (78%) reported improvements in the documentation of pain scores at their hospital. 15 (65%) strongly agreed or agreed that participation in APOP directly resulted in increased prescribing of multimodal analgesia for pain relief in postoperative patients.

**Conclusions:**

This national DUE program has facilitated the engagement and participation of a number of acute health care facilities to address issues relating to quality use of medicine. This approach has been perceived to be effective in helping them achieve improvements in patient care.

## Background

**Figure 1:**
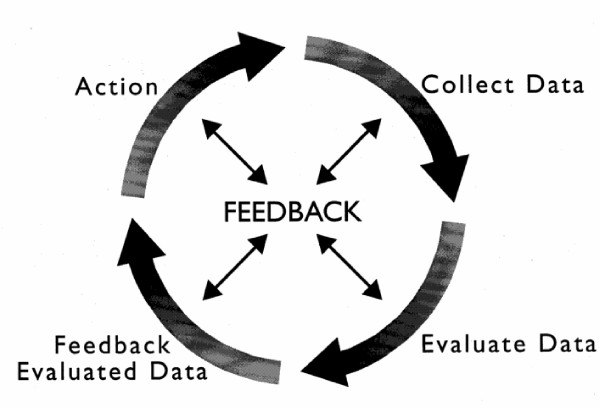
**The Drug Use Evaluation Cycle**. Reproduced from the Standards of Practice for Drug Use Evaluation in Australian Hospitals, courtesy of the Society of Hospital Pharmacists of Australia [3].

The use of medicines is the most common health-related action taken by Australians, particularly those with chronic diseases [1]. With such a broad and extensive use of medicines as part of health management, mechanisms to ensure quality use of medicines are essential [2]. Drug usage evaluation (DUE) is an evidence-based quality improvement methodology, designed to improve the quality, safety and cost-effectiveness of drug use [3,4]. DUE or drug utilisation review (DUR) involves an initial four step process of: 1. identification of a quality use of medicine problem e.g. low concordance with an accepted prescribing guideline, 2. collection of baseline data to quantify the problem, 3. feedback of results (evaluated data) to prescribers and other stakeholders, and 4. implementation of an intervention to improve practice. Ideally, this process of audit and feedback is repeated to continue to assess practice and identify gaps as in Figure 1.

Commencing in the late 1990s DUE practitioners in Australia formed state-based special interest groups that carried out multisite audits of drug use [5-7]. Participation in multisite DUE projects enabled hospitals to compare their practice with others and enabled smaller hospitals with limited resources/capacity to carry out DUE. Some of the state-based audits were sponsored by the NPS - Better choices, Better Health (NPS), an independent, non-profit organization, funded by the Australian Government Department of Health and Ageing. The NPS provides evidence-based information about medicines, and educational and behaviour change strategies for health professionals and consumers. In 2001 the NPS funded five state Therapeutic Advisory/DUE Groups to conduct separate multisite initiatives [8-12]. Topics for each state were based on local needs analysis, and the projects comprised audit and feedback and subsequent re-audit. Based on an assessment of the achievements, barriers and enablers of state-based projects, the NPS in 2003 funded and coordinated the state Therapeutic Advisory/DUE groups to work collaboratively to address quality use of medicine issues at a national level for hospitals.

Published DUEs mostly present an initial clinical audit only and tended to be localised in nature [13]. A recent systematic review of the literature demonstrated a greater chance of success in changing behaviour with regards to medication use when a number of different interventions are used [14]. The national DUE program aimed to implement the complete DUE cycle, including a baseline audit, multifaceted interventions and post-intervention audits. We describe a model of national multisite DUE and its evaluation, as illustrated by the Acute Postoperative Pain (APOP) project.

## Methods

This model utilises an established quality improvement methodology, DUE, as described previously. This involves hospitals collecting baseline audit data, feedback of evaluated data, targeted educational interventions, a repeat audit and feedback on improvements in practice. At least one complete DUE cycle is implemented during the course of each 2-year project.

### Topic selection

The state Therapeutic Advisory/DUE groups consult with their hospital members and affiliated academic units to identify a therapeutic area that may benefit from a quality improvement initiative. Key criteria for topic selection are outlined below.

The topic selected should have:

• A disease state or therapy-based focus

• Evidence-based national guidelines of best practice available

• A quantitative/qualitative gap in evidence-based practice

• Potential for engagement with hospital physicians in a shared quality agenda

• Alignment with national health priorities

• Potential for synergy with programs undertaken by other agencies

• Potential to improve patient care across the continuum of care

• Potential to link with primary healthcare providers

• A significant positive impact on patient care

• Support for sustainable quality improvement activity (capacity and skills in hospitals) and embed quality use of medicine practices into hospital systems

### Project governance

The project is managed by the national project team comprising NPS staff members (project lead, data analyst and database/website developers) and the project officer and lead from each of the state-based Therapeutic Advisory/DUE groups. State project committees are established and comprise at least one member from participating hospitals and selected key medical, nursing and pharmacy clinicians from the state with expertise in disease/therapeutic area.

**Figure 2:**
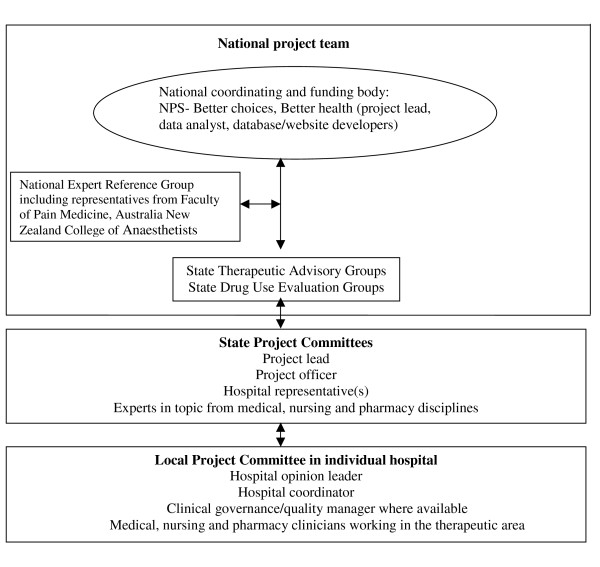
**Flow chart of the organisational structure for the national Drug Use Evaluation program for hospitals**.

A national expert reference group is established to guide the national project team and consists of members nominated by key specialist organizations. The expert reference group provides advice on gaps in evidence-based practice, project objectives, quality measures for improvement, and reviews the key messages and educational materials. For the APOP project this group consisted of members from the Australian and New Zealand College of Anaesthetists and the Faculty of Pain Medicine. The organisational structure is seen in Figure 2.

### Hospital recruitment

Australian hospitals, private and public, metropolitan and regional, are invited to participate via the state groups on a voluntary basis. A quality assurance or full ethics application is made as required by individual hospital ethics committees using the National Ethics Application Form [15] where accepted. Hospitals are provided with some financial support from NPS to assist with implementation of the project. Participating hospitals appoint a local hospital coordinator, whose responsibilities are to: liaise with the state project officer, recruit a local hospital project team, coordinate and facilitate project activities including data collection, feedback of results within the hospital, and organise educational activities and system changes where necessary. A local opinion leader, who is seen as an expert within the therapeutic area, is sought to support and endorse local project activity. A project team consisting of the local hospital coordinator, local opinion leader, medical, nursing and pharmacy clinicians working in the therapeutic area and, where available, a clinical governance/quality manager is formed.

### Data collection

Patient inclusion criteria and the dataset are determined by the national project team. The dataset includes patient demographics, relevant clinical data and prescribed medicines, information on the patients' experience of care, and information from the General Practitioner (GP) on the quality of the discharge communication. Each hospital is responsible for collecting its own data, via the inpatient medical record review, structured patient interview and GP survey.

For APOP, the dataset included information on documentation of preoperative patient education and postoperative pain assessment, postoperative analgesic prescribing and administration, monitoring and documentation of adverse effects, documentation of pain management plans at discharge, the patient's experience of pain management in hospital and following discharge, and the adequacy of communication to the GP about on-going pain management.

Data are collected and then de-identified data are entered into a specifically designed web-based database (electronic-DUE audit tool). The electronic-DUE audit tool is a secure, password protected electronic database developed by the NPS [16]. The tool generates automated feedback reports for each hospital (in real time), reporting key quality measures. Hospital de-identified data are submitted electronically via the electronic-DUE audit tool in an encrypted file to the NPS for central collation and analysis. This comparative analysis enables each hospital to compare their baseline practice and subsequent improvements to state and national data. In APOP the quality measures included documentation of pain assessment, postoperative analgesic prescribing, patient satisfaction with pain management and communication of the discharge pain management plan to the patient/carer and GP.

### Intervention

A multifaceted intervention strategy is used to deliver nationally-developed best practice guidance in the form of key messages for the project. Academic detailing (educational outreach, educational visiting) by trained detailers is the key intervention [17]. Each hospital nominates up to two staff members (medical, nursing or pharmacy) from relevant specialty areas to be trained in academic detailing skills. Two-day workshops for approximately twenty participants each, conducted by the Drug and Therapeutic Information Service (Adelaide) and NPS, are held. Participants are trained in the theory and practice of academic detailing and the evidence behind the key messages, using didactic sessions, small group work and role plays with other health professionals. The target audience for the academic detailing is determined by the hospital and includes key opinion leaders, senior and junior medical officers, nursing staff, pharmacists and other allied health members.

For the APOP project, education was addressed to medical, nursing, pharmacy and allied health staff working in surgical and anaesthetic areas. For nursing staff the focus was the use of pain and sedation scores, and for medical staff education focused on the importance of communication to the patient and GP at discharge. The evidence for prescription of multimodal analgesia, multiple anti-emetic options and prophylactic laxatives for patients prescribed opioids was emphasised to all groups.

Other interventions include point-of-prescribing prompts (e.g. reminder bookmark), promotional material (e.g. wall posters) and a feedback presentation with comparative state and national data, provided for local adaptation by each hospital. System changes are implemented at the discretion of the hospital project team.

### Evaluation

A short qualitative survey designed in consultation with state-based Therapeutic Advisory/DUE groups was undertaken by NPS between December 2009 and February 2010. The main aims of the survey were to establish: hospitals' perceptions of the effectiveness of the overall DUE methodology and the changes in procedure/prescribing/policy/clinical practice which resulted from participation in one of the three national DUE projects [18-20]. Questions were selected on the basis of eliciting quality assurance/improvement responses and to probe responses regarding the varying stages of the DUE cycle. The survey questions can be found in additional file 1. (Note: Results displayed below are for questions relating to APOP only).

Questions were embedded into the online surveying and data collection tool - Survey Monkey^(R)^. A link to the survey was emailed to a list of participating staff at hospitals, in New South Wales, Victoria, Tasmania, Queensland and South Australia. Recipients on that list were those who had an active involvement in a DUE project. The list was provided by state coordinators of the DUE projects. Responses were coded and grouped into themes according to one investigator and refined by two investigators if necessary.

## Results

### Evaluation

62 hospitals (31 principal referral, 10 large major city, 10 private, 5 large regional and remote, 3 medium major city/regional, 2 specialist women's/children's hospitals and 1 private regional hospital collaborative) from all states and territories participated in APOP. Results regarding APOP have been previously published [19].

The evaluation survey was sent to 72 nominated individuals. 37 recipients had participated in the APOP project. 23 (62%) of these 37 respondents to the evaluation survey reported being involved in the APOP project.

### Changes in practice

78% (n = 18) of the 23 APOP respondents agreed or strongly agreed that documentation of pain scores increased at their facility as a result of APOP. Responses elaborating on these changes were grouped into two themes: awareness of best practice (via in-service education) resulting in improved local practice and system change to embed best practice in care processes. System changes included a bedside pain prompt, changes in observation forms, additional posters and patient education leaflets. Some hospitals reported that the changes to observation charts had occurred at a state level.

Comments included:

'I think pain score documentation increased simply because of an increased awareness among staff of the use of pain scores as the 5th vital sign. There was a number of ward in-services given re: APOP and the use of pain scores. The inclusion of a pain score was already part of the observation chart.'

'The Nurses on the Orthopaedic ward were actively involved and really did take this to town. They started recording pain at rest and pain at movement on their Ob's charts [observation chart] where previously they had only recorded 'pain at rest '. They had the key message of 'Please remember to record your pain score' on the outside cover of each pts [patients] bedside file'

Fifteen respondents (65%) strongly agreed or agreed that participation in APOP directly resulted in increased prescribing of multimodal analgesia for pain relief in postoperative patients. Four respondents commented that the prescription of multimodal analgesia was already done well, but enhanced with increased involvement by pharmacists:

'Prescription of multimodal analgesia was already practiced in this hospital, however staff became more aware of rationale behind this and reduced co-prescription of same/similar drugs e.g. Panadeine Forte + Oxycodone'

'We were already adopting the multimodal approach but it was great to reinforce this. If for some reason it was not being used the Pharmacists were educated to question say why the regular paracetamol was not prescribed and could quite easily back it up with the literature'

One hospital reported a mandatory Junior Medical Officer pain management orientation program devised by the Acute Pain Service to improve the prescribing of analgesics. Another hospital reported on the frequent changeover of staff and the need to repeat education to sustain improvements in the prescribing of multimodal analgesia.

Ten of the respondents (43%) involved in the APOP DUE indicated that it resulted in an increased provision of postoperative pain management plans at discharge to patients and GPs. Seven (30%) indicated that there was no change at their hospital and two respondents disagreed or strongly disagreed that APOP resulted in increased pain management plans at discharge for patients. One respondent indicated that this was a time management issue for their medical staff and two respondents commented that it was either undertaken by the pharmacist or on prompting by the pharmacist. Four respondents described the development of information brochures for patients at discharge, along with development of electronic discharge summaries and discharge medication lists provided by pharmacists.

'Patient information leaflets were developed detailing drugs given at discharge. These were also faxed to GP on discharge. GP survey indicated improvement'

'Basically the Drs [doctors] always had to be prompted to make headway with this element of the project. The medication discharge summaries that we use are practically illegible ie the copy that goes with the pt to take to their local Dr. However if that pt [patients] was seen by a Pharmacist this aspect would be done well and a Medilist would be supplied too'

### DUE methodology

With regard to feedback on DUE methodology, 18 (78%) responses were received about the effectiveness of the DUE projects on improving quality use of medicines. These were grouped into a number of themes as seen in Table 1.

**Table 1:** Feedback from respondents on drug use evaluation methodology

Theme	Examples of comments
Better prescribing	*Improved analgesic prescribing by JMOs [Junior Medical Officers]*.

Improved and better documentation	*'Increase in documentation of pain scores at rest and movement due to a change in hospital paper work.'* *'Audits allow for identification of specific weaknesses in a process e.g. the lack of a pain score in the patient observation chart. It is these system changes that have the most lasting effects'*

Improvement at discharge for patients	*'Improved patient information process to understand analgesics prescribed at discharge'*

Use of benchmarking for improvement	*'It was great to have local data for feedback'*

Patient education/counselling	*'Improved provision of information to patients.'*

One respondent summed up their feeling regarding the overall DUE methodology:

'*The DUE project provided an infrastructure - data collection, analysis, intervention, and follow-up - which allows the hospital to focus on a particular area that may otherwise not happen. There is little time available in hospitals to develop all the necessary materials to conduct such quality improvement activities in isolation. Providing deadlines meant the work got done. Providing training in communication via academic detailing facilitated effective inter-professional communication around key messages. It became more about working together not about one person telling someone else what to do.'*

### Facilitators of change

Respondents were provided a list of examples that may have facilitated change in their hospital (feedback of hospital data benchmarked against state and national, involvement of Drug and Therapeutic Committees, involvement of Senior Clinician, endorsement, provision of educational resources for patients and hospital staff). Nine of the comments received focused particularly on the success that benchmarking and feedback had in facilitating change.

'Benchmarking showed improvement with implementation of education programs and development of patient information. Projects have been actively endorsed by Pharmacy & Therapeutics Committee. Provision of education for staff (both medical, nursing and allied health) has facilitated change in the hospital setting with assisted use of DUE's program templates and checklists.'

*'For a rural site like ours the state and national benchmarking was great'*.

'Benchmarking very useful'

## Discussion

We describe a unique model of a national multisite DUE program. Our model protects the privacy of the data from individual hospitals while allowing for comparisons between the participating hospital, and aggregated state and national results. Participation in a nationally coordinated program, with education resources and training provided, enables hospitals to undertake the DUE, with efficient use of local resources. Hospitals are provided with support/assistance at all stages by state project officers.

A multifaceted educational intervention strategy is employed as part of the overall methodology, allowing hospitals to select and tailor the intervention tools according to local need, to influence clinician behavior. There is an increasing international recognition of academic detailing as an important evidence-based strategy for influencing behavior change among health professionals [17,21-24]. A review by Wensing and Grol found positive effects, mainly on prescribing behavior when academic detailing was used as part of a multifaceted intervention including audit and feedback and reminders [25]. Academic detailing has been used by NPS in Australia to improve quality use of medicine in the general practice setting. In the national DUE program hospital staff were trained in skills for professional communication to influence behaviour change. These skills may be used in other local initiatives and in everyday practice.

Our model gives participating hospitals immediate access to their own results as well as enabling benchmarking with state and national results. Respondents to the evaluation survey reflected that feedback of current practice and benchmarking was particularly useful. Participation may also enable hospitals to meet local, state and national standards/indicators/accreditation requirements. Factors that facilitate implementation of a multisite project are described below.

### Factors that facilitate implementation of a multisite project

• A national coordinating body

• Experienced state project officers

• Provision of a small financial incentive

• Use of an evidence-based intervention method (academic detailing) as part of a multi-faceted intervention

• Use of an electronic data collection tool with automated feedback in real-time

• Engagement of enthusiastic hospital staff

The undertaking of a multisite DUE program has some challenges. Firstly national consensus is required in selecting a therapeutic area of interest and priority. Consensus amongst clinicians regarding appropriate management (best practice) within the selected area is also required. To facilitate national consensus, identification of best-practice guidance is required e.g. in Australia we have the Therapeutic Guidelines [26] and other national guidelines. With data being collected across many sites, the use of a manual to guide data collection is essential to ensure data integrity. In the current model submitted data are checked for obvious discrepancies; however potential misinterpretation of data elements by local data collectors is not assessed during the data collection process. This is a limitation of the multisite methodology, and could be overcome by random audits of data collection at the various sites by state project officers. The cost effectiveness of academic detailing has not been evaluated. It is unclear whether the impact of the multifaceted intervention is sustained over time [27].

In the current national DUE model financial support offered to hospitals is a contribution only, with the expectation that hospitals will provide support in kind towards project completion. Reliance on the funding from NPS to support all project related activity resulted in some hospitals being unable to complete the project as they were not able to retain project staff.

Other challenges for collaborative work include the coordination of activities across all sites within defined timeframes e.g. hospital recruitment, obtaining ethics approval and data entry/submission to enable timely reporting of state and national results. Delays in obtaining ethics approval, variation in rate of presentation of eligible patients across participating sites, management of project workload and staff leave may influence the ability of the project teams in meeting key milestones/deadlines.

A challenge for most quality improvement activities at a hospital level is the rotation of junior hospital staff to other clinical areas. Repeated education is therefore required as new staff are rotated through the targeted clinical area. This was highlighted by comments from the evaluation survey. Conversely rotating staff take the learnings with them to other clinical areas which may facilitate the spread of the educational messages to a wider audience.

To promote sustainability and spread of improvements achieved, the NPS provides the electronic DUE tool as a stand-alone version freely available online after the completion of the project [28]. Hospitals are encouraged to undertake further audits of their practice and continue quality improvement.

## Conclusion

This national program has facilitated the participation of a substantial number of hospitals to undertake DUE, and deliver standardized and consistent key educational messages across Australia. The model has been perceived by hospitals to be effective in helping hospitals achieve improvements in patient care.

## Competing interests

The authors declare that they have no competing interests.

## Authors' contributions

LKP, AW, DJM and MRB conceived the project. LKP, AW, DJM, MRB, SR participated in its design and coordination. SR collected the data. LKP, AW, DJM, MRB, SR drafted the manuscript. All authors read and approved the final manuscript.

## Pre-publication history

The pre-publication history for this paper can be accessed here:

http://www.biomedcentral.com/1472-6963/11/206/prepub

## Supplementary Material

Additional file 1 **Multicentre Drug Use Evaluation survey**. Multicentre DUE survey questions.doc. Click here for file
