# Supplementary material for: Implementation and evaluation of a multisite drug usage evaluation program across Australian hospitals - a quality improvement initiative
Source: BMC Health Serv Res. 2011 Aug 29;11:206. doi: 10.1186/1472-6963-11-206 (PMC3182891; doi:10.1186/1472-6963-11-206)
Supplement: Additional file 1 — Multicentre Drug Use Evaluation survey. Multicentre DUE survey questions.doc. [file 1472-6963-11-206-S1.DOC]

**Multicentre Drug Use Evaluation survey**

**The purpose of this survey is to seek/evaluate your impressions relating to a Multicentre Drug Use Evaluation you may have been involved with at your hospital**

Multicentre Drug Use Evaluation (DUE) activities in hospitals have been conducted nationally in collaboration with state-based Therapeutic Advisory Groups (TAGs) and DUE Groups from New South Wales, Tasmania, Victoria, South Australia, and Queensland through funding and support provided by the National Prescribing Service (NPS) since 2004.

These projects utilise established quality improvement methodology, DUE, that involves hospitals collecting baseline data, feedback of evaluated data, targeted educational interventions, a repeat data collection and feedback on the success of the interventional strategies. At least one complete DUE cycle is implemented during the course of the 2-year project. This quality improvement methodology has been applied successfully to a number of diverse national initiatives as follows:

**DUE 1: Community-Acquired Pneumonia Towards Improving Outcomes Nationally (CAPTION) project (2004-2006)**

CAPTION was a national quality improvement project to promote and increase the uptake of the Guidelines community-acquired pneumonia recommendations in Australian hospital emergency departments, specifically aiming to improve appropriate antibiotic use and the use of a pneumonia severity assessment tool, the Pneumonia Severity Index (PSI). The project promoted the 2003 Therapeutic Guidelines: Antibiotic for the Management of community-acquired pneumonia.

**DUE 2: Acute Postoperative Pain (APOP) project (2006-2007)**

The APOP project aimed to promote assessment of pain, safe and effective analgesic prescribing and communication of pain management plans at discharge to patients and general practitioners (GPs).

**DUE 3: Discharge Management of Acute Coronary Syndromes (DMACS) project (2008-2009)**

The aims of the DMACS project are to optimise prescription of cardiovascular medications, improve education on lifestyle modifications, and increase communication with patients and general practitioners (GP). The project promoted the 2006 National Heart Foundation Guidelines for the Management of Acute Coronary Syndromes.

**Questions:**

1. Indicate which personnel were involved in implementing the DUE at your hospital. (Mark all that apply)

Nurse  Hospital pharmacist

Medical Registrar  Quality manager/coordinator

Medical Specialist  Other, please specify....................

Director of Nursing

1. Indicate who was the main ‘driver’ or ‘champion’ for the DUE program in your hospital? (Mark all that apply)

Nurse  Hospital pharmacist

Medical Registrar  Quality manager/coordinator

Medical Specialist  Unsure

Director of Nursing  Other please specify....................

1. Did you complete the DUE project called CAPTION at your facility?

Yes – Please complete the following questions

No – Please go to question 4.

Unsure – Please go to question 4.

Please circle the appropriate response

1. CAPTION was useful in improving appropriate antibiotic use for community-acquired pneumonia at your hospital

Strongly agree / Agree / No change / Disagree / Strongly Disagree / Don’t know

1. If you agreed or strongly agreed, please provide an example of how CAPTION was useful in improving appropriate antibiotic use for the management of pneumonia.

____________________________________________________________________________________________________________________________________

1. CAPTION resulted in an increase in the use of the pneumonia severity index assessment tool

Strongly agree / Agree / No change / Disagree / Strongly Disagree / Don’t know

1. If you agreed or strongly agreed, please provide an example of how CAPTION increased the use of a pneumonia severity index assessment tool. ____________________________________________________________________________________________________________________________________
2. Overall, how effective was CAPTION in improving antibiotic use in the management of pneumonia at your hospital?

Very effective / Effective / Slightly effective / Not at all effective / Don’t know

1. Did you complete the DUE project called APOP (Acute Post Operative Pain) at your facility?

Yes – Please complete the following questions

No – Please go to question 5.

Unsure – Please go to question 5.

1. APOP resulted in an increase in the use of pain assessment tool to measure pain in patients following surgery at your facility?

Strongly agree / Agree / No change / Disagree / Strongly Disagree / Don’t know

1. If you agreed or strongly agreed, please describe how APOP was responsible for the increase in the assessment of postoperative pain in your facility.

____________________________________________________________________________________________________________________________________

1. Participation in APOP directly resulted in improvements in safe and effective postoperative analgesic use for postoperative patients?

Strongly agree / Agree / No change / Disagree / Strongly Disagree / Don’t know

1. If you agreed or strongly agreed, please describe how this was achieved.

____________________________________________________________________________________________________________________________________

1. APOP resulted in an increase in communication of postoperative pain management plans at discharge to patients and general practitioners (GPs)?

Strongly agree / Agree / No change / Disagree / Strongly Disagree / Don’t know

1. If you agreed or strongly agreed, please describe how patients were provided pain management plans and what follow up procedures were carried out with GPs.

____________________________________________________________________________________________________________________________________

1. Did you complete the DUE project called DMACS at your facility?

Yes – Please complete the following questions

No – Please go to question 6.

Unsure – Please go to question 6.

1. Describe how prescribing of cardiovascular medications at discharge changed at your hospital as a result of DMACS?

____________________________________________________________________________________________________________________________________

1. Overall, how effective was DMACS in improving cardiovascular medications use in your hospital?

Very effective / Effective / Slightly effective / Not at all effective / Don’t know

1. a) Were there any local guidelines for prescribing (or policy/procedures/protocols) introduced as a result of participation in one of the DUE projects.

Yes  No

If yes, please give an example of the guidelines which were introduced.

_____________________________________________________________________

b) Describe how these guidelines have changed practice in your facility? Please provide at least one example.

__________________________________________________________________________________________________________________________________________

1. If the DUE was found to be effective in improving medication use, please describe how and/or why the DUE was effective. Please provide at least one example.

__________________________________________________________________________________________________________________________________________

1. Were there any modifications or system changes *other* than medication use which occurred in your hospital? (e.g. sustained use of education materials and assessment tools provided (pneumonia severity index, pain scale/s, inclusion of pain scores in patient observation chart), patient hand-outs/information (Managing pain, Heart Foundation wallet card on “Angina & Heart Attack warning signs - what to do”); modification to discharge processes using checklist and templates provided

Yes, please describe______________________________________________

No

Unsure

1. What, if any aspect of the DUE facilitated change in your hospital (e.g. electronic (e-DUE) audit tools, feedback of hospital audit data benchmarked against state and national, ability to measure size of change compared with baseline audit following education, involvement of Drugs and Therapeutic Committees, Quality & Safety unit, Leadership and involvement of Senior Clinician, provision of educational resources for patients and hospital staff?

Yes, please describe______________________________________________

No

Unsure

1. Were there any other Quality and Safety initiatives were undertaken in the hospital concurrently with the DUE project?

Yes, please describe what these initiatives were and how they may have reinforced or confounded the DUE. ________________________________________

_____________________________________________________________________

No

Unsure

**For the following four statements, indicate the five ‘drivers’ of the DUE program** *(this question will appear as a matrix)*

1. Would be likely to motive your hospital/Area Health Service to be involved in future quality programs for medicines:
2. Would be likely to motive national and/or State Health Authorities to be involved in future quality programs for medicines:
3. Would be likely to result in improvements being embedded in your hospital from a quality use of medicines program:
   1. reduced demand on hospital/department budget
   2. reduced in-patient drug costs
   3. reduce Full-time equivalents employed
   4. death avoided
   5. reducing length of stay
   6. admission/readmission avoided
   7. adverse drug reactions avoided
   8. acheivement of mandatory standard (e.g. The Australian Council on Healthcare Standards (ACHS) EQuIP - accreditation standards/performance measures)
   9. improving communication between hospital & GP
   10. improved patient satisfaction with hospital
   11. reduced Pharmaceutical Benefit Schedule drug costs
   12. acheivement of non-mandatory standard (e.g. ACHS EQuIP standards)
   13. staff education
   14. don’t know
   15. other, please specify
4. What model would your hospital agree to continue to participate in for an ongoing quality improvement for medicine use? (Mark all that apply)

Model 1: Continuum of care model is an initiative which uses hospital admission to influence chronic disease management in the community. Patients receiving specified medication (or with specified conditions) where there are recognised opportunities to improve the implementation of evidence based treatment strategies, improve patient safety, reduce hospital admissions and / or reduce drug costs; will be targeted for review.

Model 2: A model using existing DUE tools, that engages with state health/ medicine committee to roll out a state DUE program.Collaboration across states and territories and national organisations to improve Quality Use of Medicine (QUM) using DUE methodology.

Model 3: A web-based, National QUM reporting tool used to measure QUM performance with the capacity to benchmark hospitals (across states and territories), includes discussion forums with the experts and an interventional 'tool exchange' as some of its key features.

Model 4: A QUM initiative that enables hospitals/Area Health Services to monitor new drug/s uptake

1. How likely would your hospital be to participate in a new model of quality improvement for medicine use in the future

Very likely / Likely / Unlikely / Very unlikely / Can’t say

1. Would you recommend NPS DUE to your colleagues at your hospital?

Yes

No

Maybe

**THANK YOU FOR YOUR PARTICIPATION**
